# Supplementary material for: BMP4 drives primed to naïve transition through PGC-like state
Source: Nat Commun. 2022 May 19;13:2756. doi: 10.1038/s41467-022-30325-4 (PMC9120449; doi:10.1038/s41467-022-30325-4)
Supplement: Supplementary file 1 — Supplementary information [file 41467_2022_30325_MOESM1_ESM.pdf]

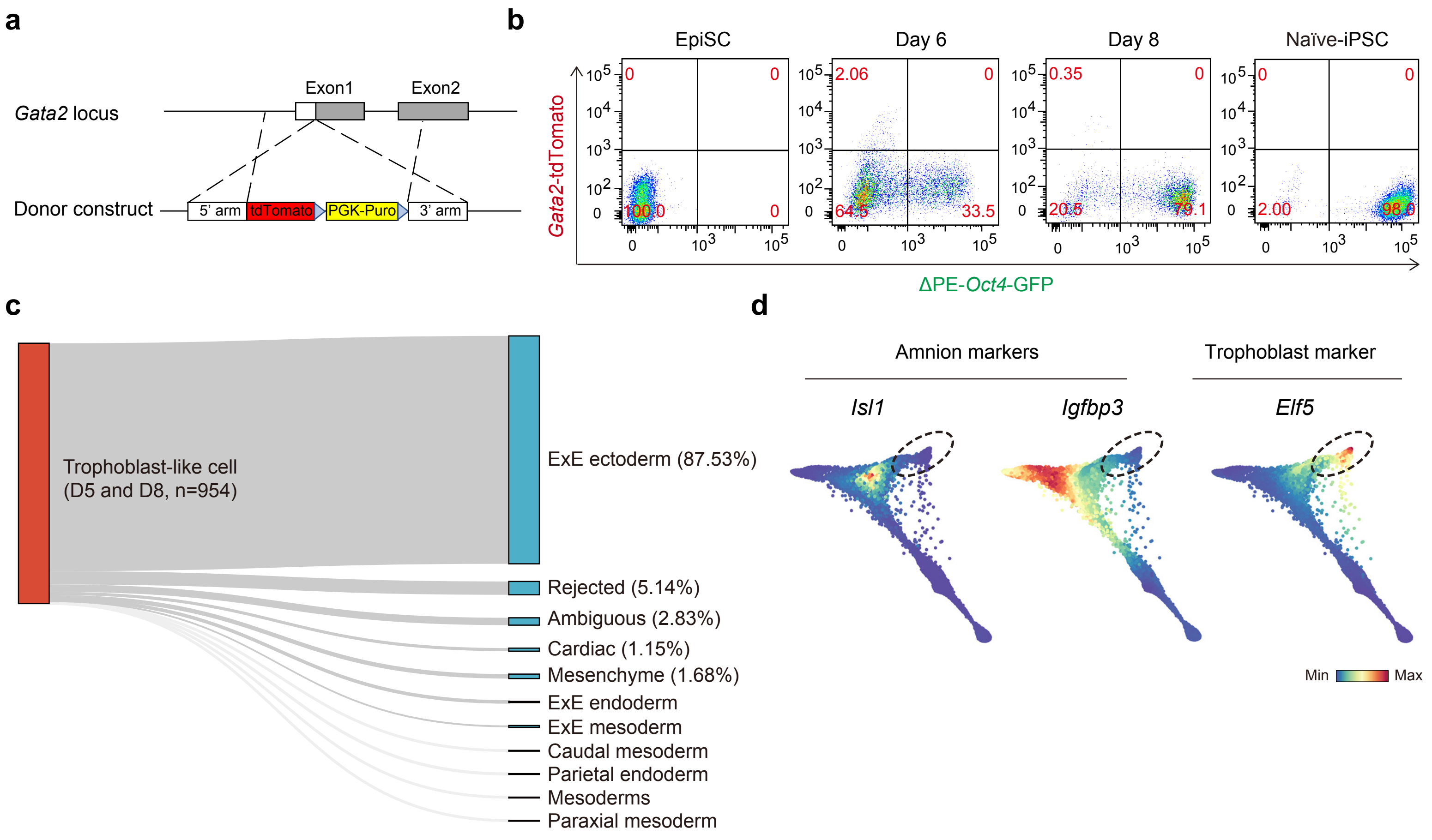
a
Gata2 locus
Exon1
Exon2
Donor construct
5' arm
tdTomato
PGK-Puro
3' arm
b
EpiSC
Day 6
Day 8
Naïve-iPSC
Gata2-tdTomato
ΔPE-Oct4-GFP
c
Trophoblast-like cell
(D5 and D8, n=954)
ExE ectoderm (87.53%)
Rejected (5.14%)
Ambiguous (2.83%)
Cardiac (1.15%)
Mesenchyme (1.68%)
ExE endoderm
ExE mesoderm
Caudal mesoderm
Parietal endoderm
Mesoderms
Paraxial mesoderm
d
Amnion markers
Trophoblast marker
Isl1
Igfbp3
Elf5
Min
Max

Supplementary Fig. 1 Generation of trophoblast-like cells during BiPNT. a Targeting strategy of generating *Gata2*-tdTomato knock-in reporter EpiSCs. **b** FACS analysis of ΔPE-*Oct4*-GFP or *Gata2*-tdTomato positive cells during BiPNT. Data shown are from 1 of 3 independent experiments. **c** Sankey plot comparing the trophoblast-like cells with the reference mouse gastrulation single-cell dataset from Pijuan-Sala, et al., Nature, 2019. **d** Plots showing the expression patterns of amnion markers *Isl1*, *Igfbp3* and trophoblast marker *Elf5* during BiPNT. *Elf5* positive trophoblast-like cells were highlighted by black dotted line.

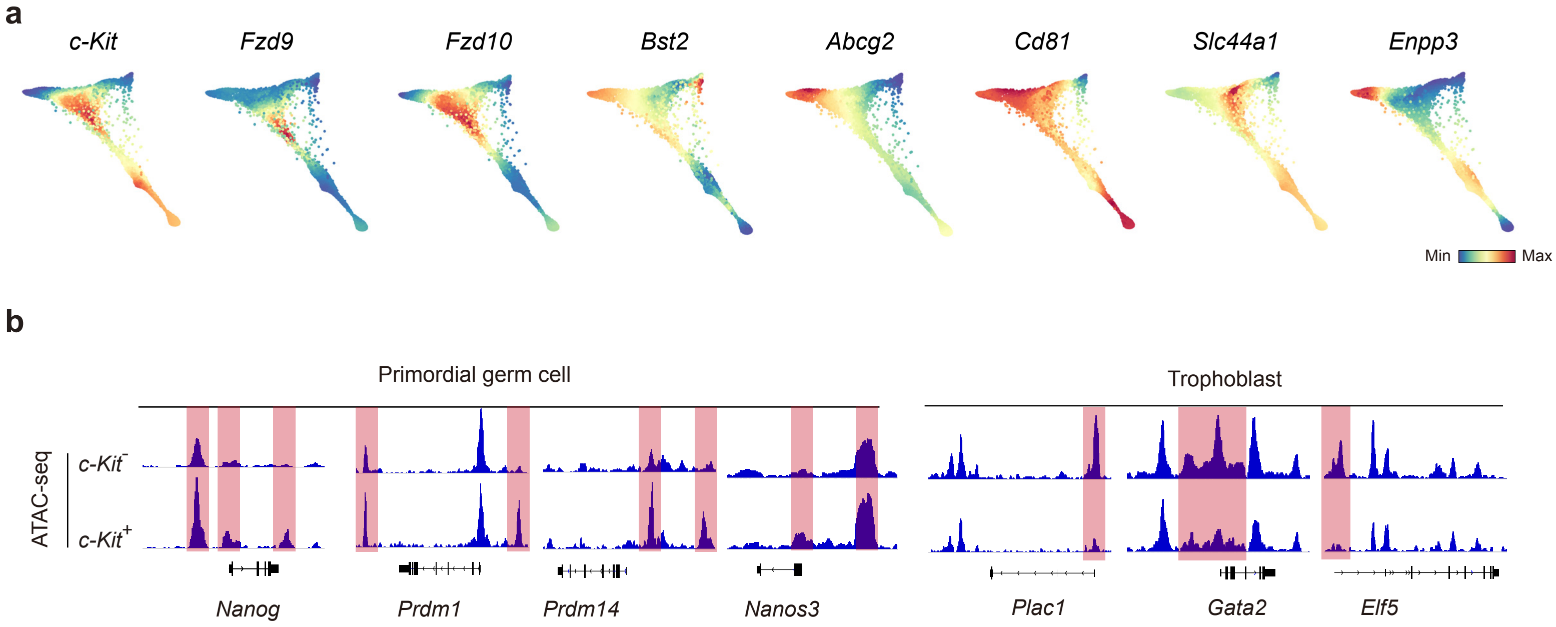
a
c-Kit
Fzd9
Fzd10
Bst2
Abcg2
Cd81
Slc44a1
Enpp3
Min
Max
b
Primordial germ cell
Trophoblast
ATAC-seq
c-Kit⁻
c-Kit⁺
Nanog
Prdm1
Prdm14
Nanos3
Plac1
Gata2
Elf5

Supplementary Fig. 2 Cell surface marker *c-Kit* indicates the early naïve branch. a Candidates of cell surface markers for naïve branch. **b** Representative loci for ATAC-seq peaks in *c-Kit* positive and negative cell samples at Day 3 of BiPNT.

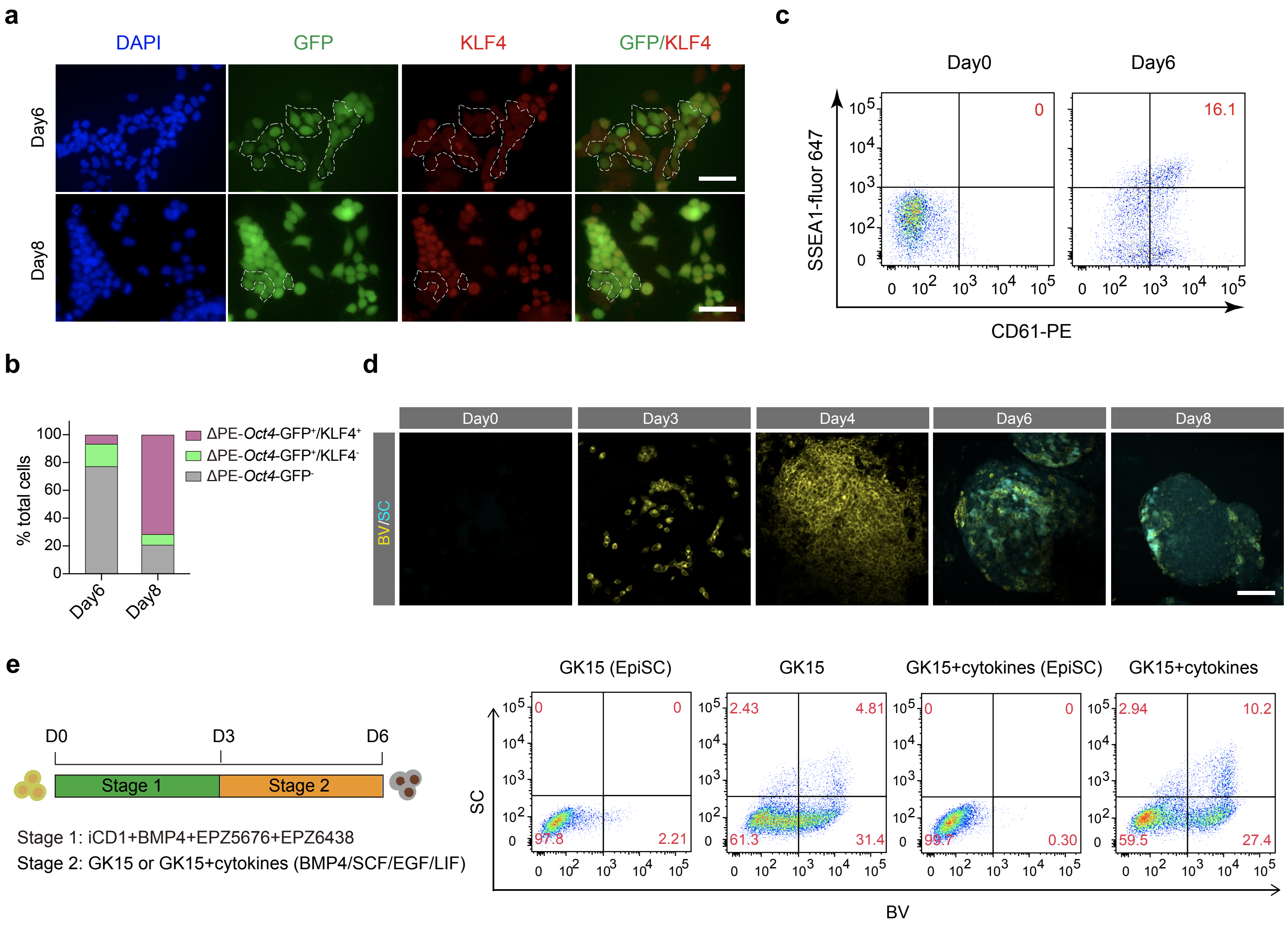

a
DAPI
GFP
KLF4
GFP/KLF4
Day6
Day8
b
% total cells
100
80
60
40
20
0
Day6
Day8
ΔPE-Oct4-GFP+/KLF4+
ΔPE-Oct4-GFP+/KLF4-
ΔPE-Oct4-GFP-
c
Day0
Day6
SSEA1-fluor 647
CD61-PE
0
16.1
d
Day0
Day3
Day4
Day6
Day8
BV/SC
e
D0
D3
D6
Stage 1
Stage 2
Stage 1: iCD1+BMP4+EPZ5676+EPZ6438
Stage 2: GK15 or GK15+cytokines (BMP4/SCF/EGF/LIF)
GK15 (EpiSC)
GK15
GK15+cytokines (EpiSC)
GK15+cytokines
SC
BV
0
0
97.8
2.21
2.43
4.81
61.3
31.4
0
0
99.7
0.30
2.94
10.2
59.5
27.4

Supplementary Fig. 3 Activation of PGC program during the naïve branch. a Immunofluorescence analysis of KLF4 in ΔPE-*Oct4*-GFP positive cells at Day 6 and 8 of BiPNT. Scale bars, 50 μm. **b** Percentage of indicated cells at Day 6 and 8 of BiPNT. **c** FACS analysis for surface markers SSEA1 and CD61 in Day 6 cells of BiPNT. **d** Representative images of BV or SC cells during BiPNT. Scale bars, 50 μm. The experiments were repeated independently three times with similar results. **e** Left: schematic of experimental approach. Right: FACS analysis of BVSC induction at Day 6 when stage 2 medium was changed to GK15 (GMEM+15% KSR) medium with or without cytokines (BMP4, EGF, SCF and LIF). EpiSC cultured in GK15 medium with or without cytokines for 3 days was as control. Source data are provided as a Source Data file.

a

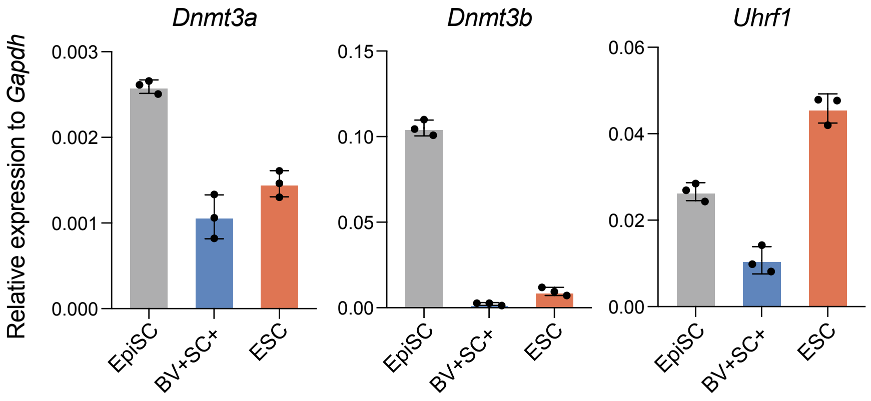

b

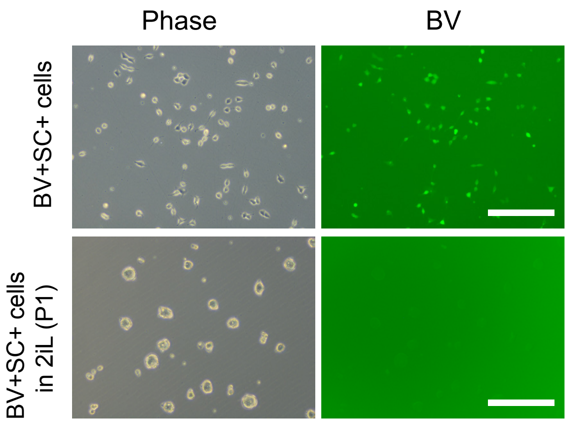

c

Injected cells	Transfered embryos	Live pups	Chimeras (%)
BV+SC+	30	8	1 (12.5)
BV+SC+ in 2iL (P1)	40	9	6 (66.7)

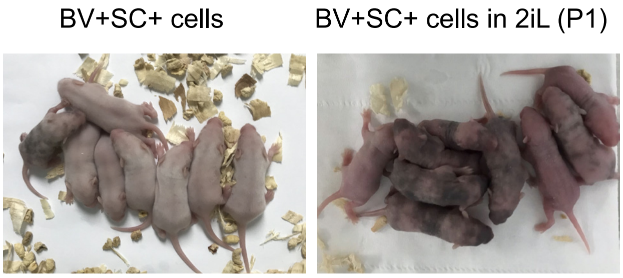

d

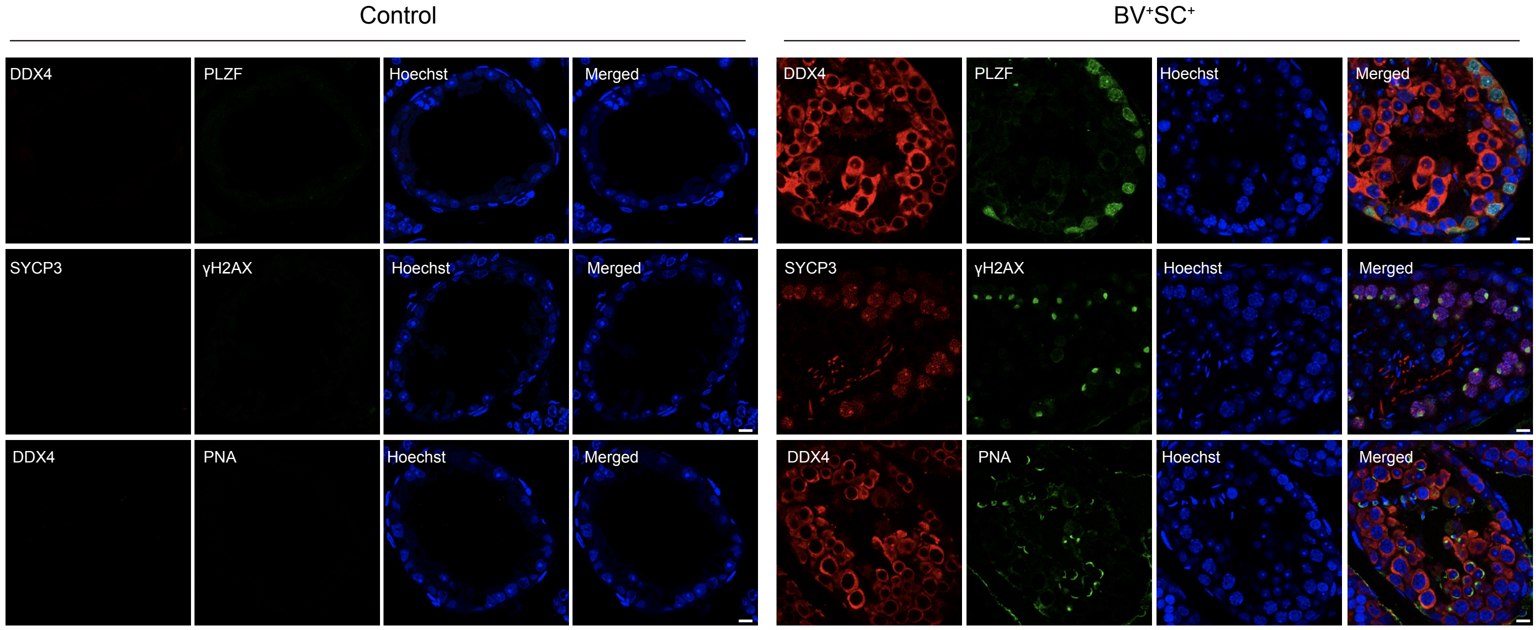

Supplementary Fig. 4 Characterization of BV$^+$SC$^+$ cells. a Expression of *Dnmt3a*, *Dnmt3b* and *Uhrf1* in EpiSC, BV$^+$SC$^+$ cells and ESC. Data are mean ± s.d., n=3 independent experiments. **b** BV is expressed in sorted BV$^+$SC$^+$ cells but not in formed colonies after incubation with 2iL medium for 1 passage (P1). Scale bars, 250 μm. **c** Capacity of BV$^+$SC$^+$ cells or BV$^+$SC$^+$ cells cultured with 2iL medium for 1 passage (P1) to contribute to chimeras. **d** Representative images showing the spermatogonia (DDX4 and PLZF double-positive cells), spermatocytes (SYCP3 and γH2AX double-positive cells) and spermatids (DDX4 and PNA double-positive cells) in testis sections from *W/W^v* mice that was transplanted with BV$^+$SC$^+$ cells, but not from control *W/W^v* mice. Scale bars, 10 μm. Source data are provided as a Source Data file.

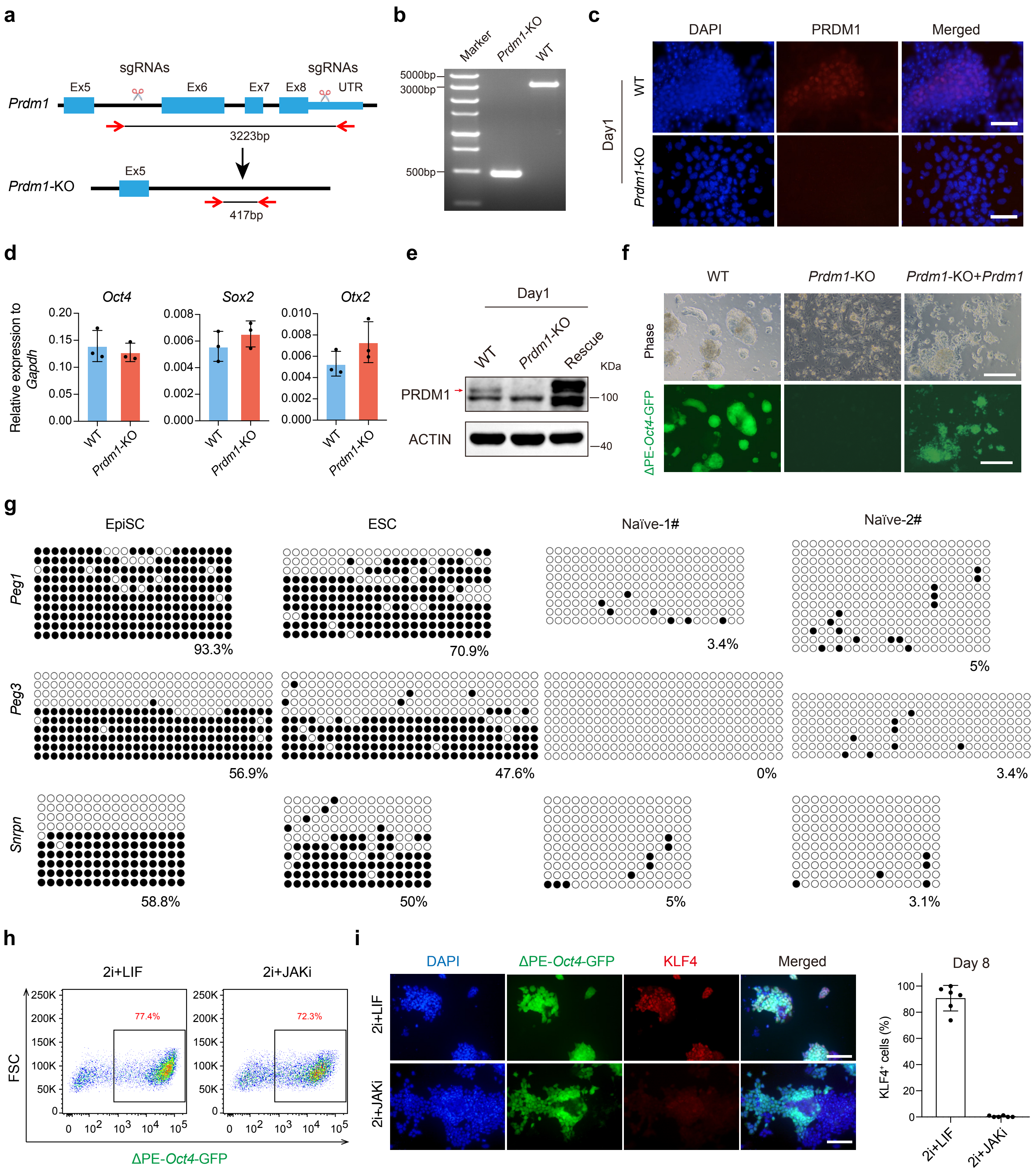
a
sgRNAs
sgRNAs
Ex5
Ex6
Ex7
Ex8
UTR
Prdm1
3223bp
Prdm1-KO
417bp
b
Marker
Prdm1-KO
WT
5000bp
3000bp
500bp
c
DAPI
PRDM1
Merged
Day1
WT
Prdm1-KO
d
Relative expression to Gapdh
Oct4
Sox2
Otx2
WT
Prdm1-KO
e
Day1
WT
Prdm1-KO
Rescue
KDa
PRDM1
ACTIN
100
40
f
WT
Prdm1-KO
Prdm1-KO+Prdm1
Phase
ΔPE-Oct4-GFP
g
EpiSC
ESC
Naïve-1#
Naïve-2#
Peg1
93.3%
70.9%
3.4%
5%
Peg3
56.9%
47.6%
0%
3.4%
Snrpn
58.8%
50%
5%
3.1%
h
2i+LIF
2i+JAKi
77.4%
72.3%
FSC
ΔPE-Oct4-GFP
i
DAPI
ΔPE-Oct4-GFP
KLF4
Merged
2i+LIF
2i+JAKi
Day 8
KLF4+ cells (%)

Supplementary Fig. 5 *Prdm1* deficient cells fail to enter PGCLC and naïve pluripotent state. **a** Scheme showing the strategy used to generate *Prdm1* knockout EpiSCs. **b** *Prdm1*-KO EpiSCs were genotyped using primers indicated in **a**. **c** Immunofluorescence analysis confirms the deficiency of PRDM1 protein at Day 1 *Prdm1*-KO cells of BiPNT. Scale bar, 50 μm. **d** RT-qPCR analysis for expression of primed pluripotency genes in WT and *Prdm1*-KO EpiSCs. Data are mean ± s.d., n=3 independent experiments. **e** Western blot analysis of PRDM1 protein in WT, *Prdm1*-KO and rescue cells at Day 1 of BiPNT. **f** *Prdm1* knockout failed to induce ΔPE-*Oct4*-GFP$^+$ cells. Scale bars, 250 μm. The experiments were repeated independently three times with similar results. **g** Bisulfite sequencing of *Peg1*, *Peg3* and *Snrpn* imprinted loci in EpiSC, ESC and two naïve colonies derived from BiPNT. **h** FACS analysis of ΔPE-*Oct4*-GFP$^+$ cells at Day 8 of BiPNT under 2i+LIF or 2i+JAKi (Jak inhibitor I, 5 μM) condition. **i** Left: Immunofluorescence analysis of KLF4 in ΔPE-*Oct4*-GFP$^+$ cells derived from the conditions as **h**. Scale bars, 100 μm. Right: Percentage of KLF4$^+$ cells in ΔPE-*Oct4*-GFP$^+$ cells. Data are mean ± s.d., n=6 microscope fields. The experiments in **b**-**c**, **e**-**f** were performed twice with similar results. Source data are provided as a Source Data file.

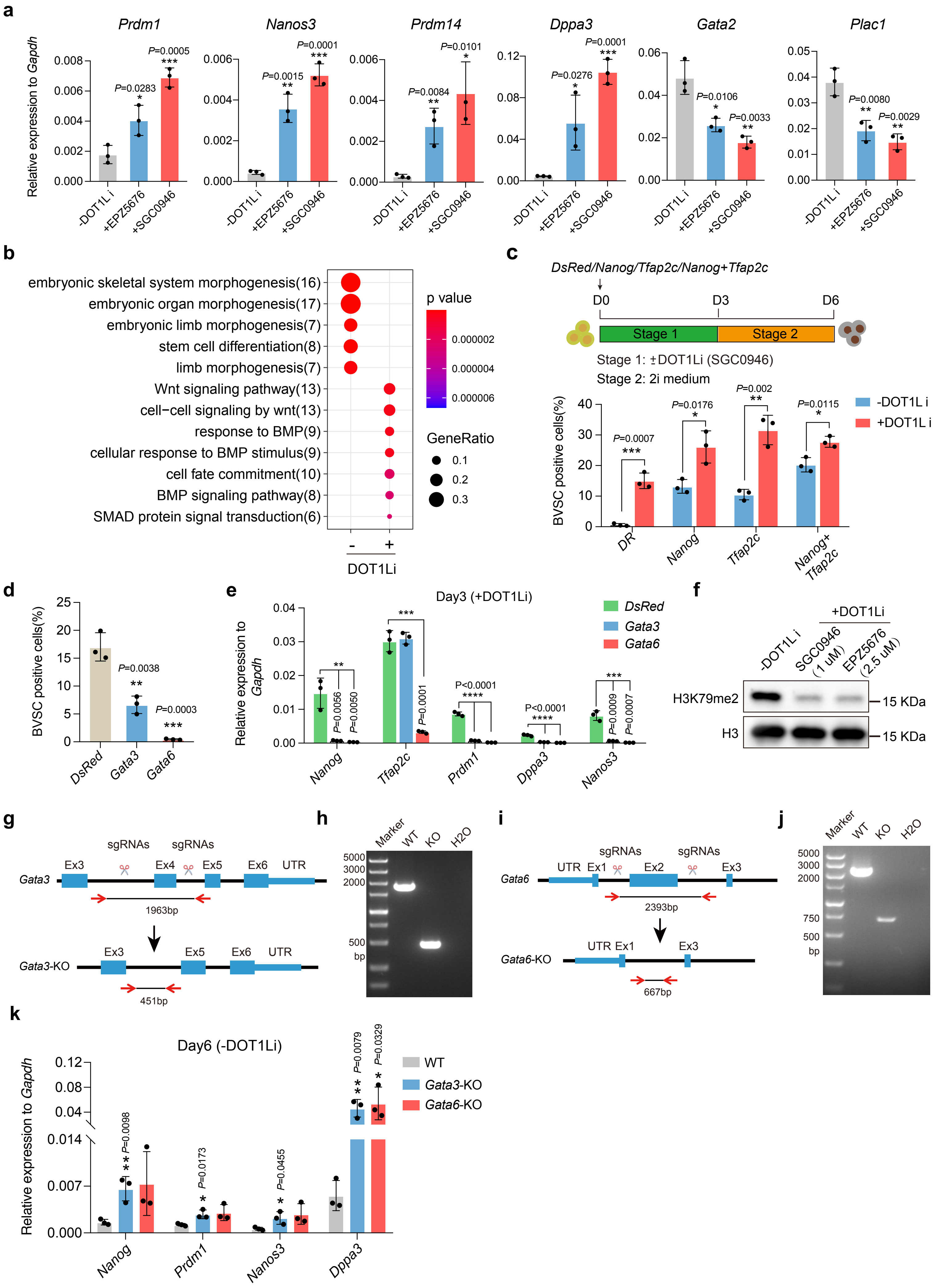

a
Prdm1
Nanos3
Prdm14
Dppa3
Gata2
Plac1
Relative expression to Gapdh
-DOT1L i
+EPZ5676
+SGC0946
P=0.0283
P=0.0005
P=0.0015
P=0.0001
P=0.0084
P=0.0101
P=0.0276
P=0.0001
P=0.0106
P=0.0033
P=0.0080
P=0.0029
b
embryonic skeletal system morphogenesis(16)
embryonic organ morphogenesis(17)
embryonic limb morphogenesis(7)
stem cell differentiation(8)
limb morphogenesis(7)
Wnt signaling pathway(13)
cell−cell signaling by wnt(13)
response to BMP(9)
cellular response to BMP stimulus(9)
cell fate commitment(10)
BMP signaling pathway(8)
SMAD protein signal transduction(6)
p value
0.000002
0.000004
0.000006
GeneRatio
0.1
0.2
0.3
DOT1Li
c
DsRed/Nanog/Tfap2c/Nanog+Tfap2c
D0
D3
D6
Stage 1
Stage 2
Stage 1: ±DOT1Li (SGC0946)
Stage 2: 2i medium
BVSC positive cells(%)
P=0.0007
P=0.0176
P=0.002
P=0.0115
-DOT1L i
+DOT1L i
DR
Nanog
Tfap2c
Nanog+ Tfap2c
d
BVSC positive cells(%)
P=0.0038
P=0.0003
DsRed
Gata3
Gata6
e
Day3 (+DOT1Li)
Relative expression to Gapdh
DsRed
Gata3
Gata6
P=0.0056
P=0.0050
P=0.0001
P<0.0001
P<0.0001
P=0.0009
P=0.0007
Nanog
Tfap2c
Prdm1
Dppa3
Nanos3
f
+DOT1Li
-DOT1L i
SGC0946 (1 uM)
EPZ5676 (2.5 uM)
H3K79me2
H3
15 KDa
15 KDa
g
sgRNAs
sgRNAs
Ex3
Ex4
Ex5
Ex6
UTR
Gata3
1963bp
Gata3-KO
451bp
h
Marker
WT
KO
H2O
5000
3000
2000
500
bp
i
sgRNAs
sgRNAs
UTR
Ex1
Ex2
Ex3
Gata6
2393bp
Gata6-KO
667bp
j
Marker
WT
KO
H2O
5000
3000
2000
750
500
bp
k
Day6 (-DOT1Li)
Relative expression to Gapdh
WT
Gata3-KO
Gata6-KO
P=0.0098
P=0.0173
P=0.0455
P=0.0079
P=0.0329
Nanog
Prdm1
Nanos3
Dppa3

Supplementary Fig. 6 DOT1L inhibition promotes the generation of PGCLCs. a RT-qPCR analysis for expression of indicated genes at Day 6 of BiPNT with or without treatment of DOT1Li (EPZ5676 and SGC0946). Data are mean ± s.d., two-tailed, unpaired student's *t*-test, n=3 independent experiments. *$p < 0.05$, **$p < 0.01$, ***$p < 0.001$. **b** GO analysis of the genes that are down-regulated or up-regulated by DOT1Li (SGC0946). **c** Upper: schematic of experimental approach. Lower: percentage of BVSC positive cells at Day 6 induced in *DsRed*, *Nanog*, *Tfap2c* or *Nanog*+*Tfap2c* over-expressed cells with or without DOT1Li (SGC0946) treatment. Data are mean ± s.d., two-tailed, unpaired student's *t*-test, n=3 independent experiments. *$p < 0.05$, **$p < 0.01$, ***$p < 0.001$. **d** Percentage of BVSC positive cells at Day 6 induced in *DsRed*, *Gata3* or *Gata6* over-expressed cells with DOT1Li (SGC0946) treatment. Data are mean ± s.d., two-tailed, unpaired student's *t*-test, n=3 independent experiments. **$p < 0.01$, ***$p < 0.001$. **e** RT-qPCR analysis for expression of PGC related genes at Day 3 of DOT1Li (SGC0946) mediated BiPNT after over-expression of *Gata3* or *Gata6*. Data are mean ± s.d., two-tailed, unpaired student's *t*-test, n=3 independent experiments. **$p < 0.01$, ***$p < 0.001$. **f** DOT1Li (EPZ5676 and SGC0946) resulted in the reduction of H3K79me2 level as measured by western blot. **g** Scheme showing the strategy used to generate *Gata3*-KO EpiSC. **h** *Gata3*-KO EpiSC was genotyped using primers indicated in **g**. **i** Scheme showing the strategy used to generate *Gata6*-KO EpiSC. **j** *Gata6*-KO EpiSC was genotyped using primers indicated in **i**. **k** RT-qPCR analysis for expression of PGC related genes at Day 6 of BiPNT in *Gata3*-KO or *Gata6*-KO cells. Data are mean ± s.d., two-tailed, unpaired student's *t*-test, n=3 independent experiments. *$p < 0.05$, **$p < 0.01$. The experiments in **f**, **h**, **j** were performed twice with similar results. Source data are provided as a Source Data file.

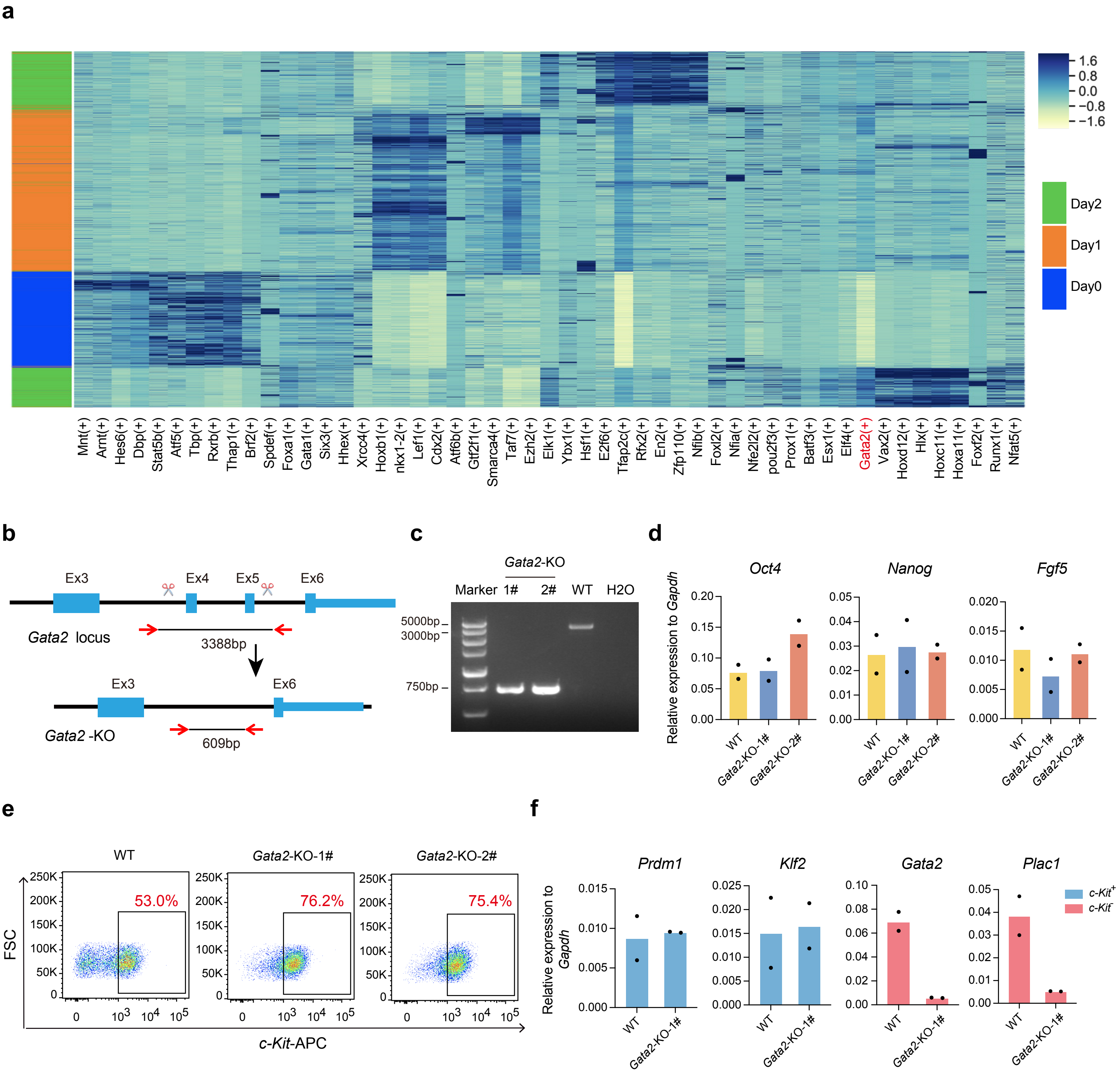
a
1.6
0.8
0.0
−0.8
−1.6
Day2
Day1
Day0
Mnt(+)
Arnt(+)
Hes6(+)
Dbp(+)
Stat5b(+)
Atf5(+)
Tbp(+)
Rxrb(+)
Thap1(+)
Brf2(+)
Spdef(+)
Foxa1(+)
Gata1(+)
Six3(+)
Hhex(+)
Xrcc4(+)
Hoxb1(+)
nkx1-2(+)
Lef1(+)
Cdx2(+)
Atf6b(+)
Gtf2f1(+)
Smarca4(+)
Taf7(+)
Ezh2(+)
Elk1(+)
Ybx1(+)
Hsf1(+)
E2f6(+)
Tfap2c(+)
Rfx2(+)
En2(+)
Zfp110(+)
Nfib(+)
Foxl2(+)
Nfia(+)
Nfe2l2(+)
pou2f3(+)
Prox1(+)
Batf3(+)
Esx1(+)
Elf4(+)
Gata2(+)
Vax2(+)
Hoxd12(+)
Hlx(+)
Hoxc11(+)
Hoxa11(+)
Foxf2(+)
Runx1(+)
Nfat5(+)
b
Ex3
Ex4
Ex5
Ex6
Gata2 locus
3388bp
Ex3
Ex6
Gata2 -KO
609bp
c
Gata2-KO
Marker
1#
2#
WT
H2O
5000bp
3000bp
750bp
d
Relative expression to Gapdh
Oct4
Nanog
Fgf5
WT
Gata2-KO-1#
Gata2-KO-2#
e
WT
Gata2-KO-1#
Gata2-KO-2#
53.0%
76.2%
75.4%
FSC
c-Kit-APC
f
Relative expression to Gapdh
Prdm1
Klf2
Gata2
Plac1
c-Kit+
c-Kit-
WT
Gata2-KO-1#

Supplementary Fig. 7 Deficiency of *Gata2* arrests trophoblast fate, but promotes PGCLCs.

a Regulon activity analyzed by pySCENIC during first three days, Day 0, Day 1 and Day 2. **b** Scheme showing the strategy used to generate *Gata2* knockout EpiSCs. **c** *Gata2* knockout cells were genotyped using primers indicated in **b**. The experiments were performed twice with similar results. **d** RT-qPCR analysis for expression of primed pluripotency related genes in WT and *Gata2*-KO EpiSCs. Data are mean ± s.d., n=2 independent experiments. **e** *Gata2*-KO cells were sorted for *c-Kit* with FACS. **f** RT-qPCR analysis for expression of indicated genes in *c-Kit*$^{+}$ or c-*Kit*$^{-}$ cells sorted from WT and *Gata2* knockout samples. Data are mean ± s.d., n=2 independent experiments. Source data are provided as a Source Data file.

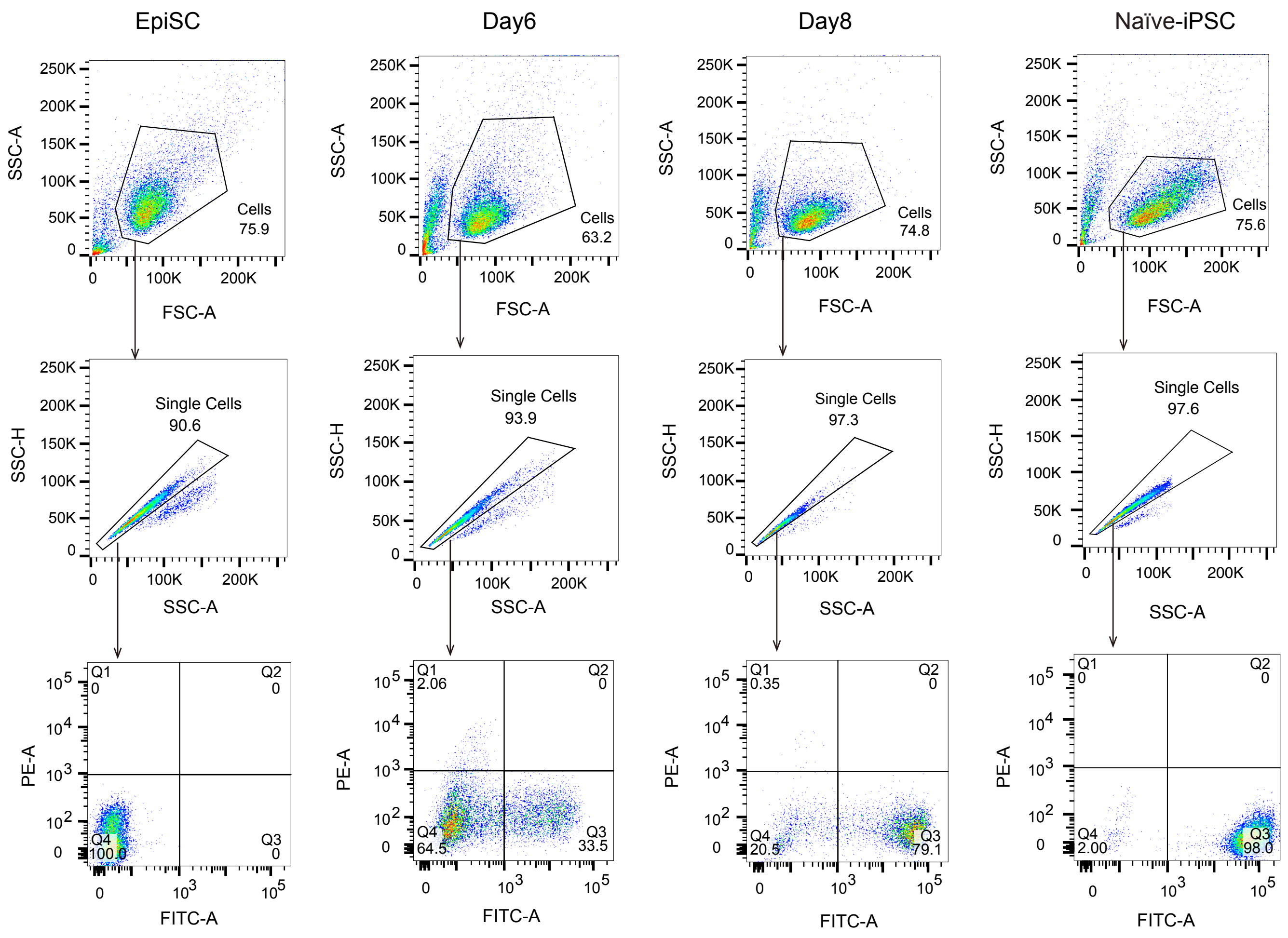

Supplementary Fig. 8 The sequential gating strategy for FACS analysis in Supplementary Fig. 1b. FACS analysis of ΔPE-*Oct4*-GFP or *Gata2*-tdTomato positive cells during BiPNT.

Original uncropped western blots

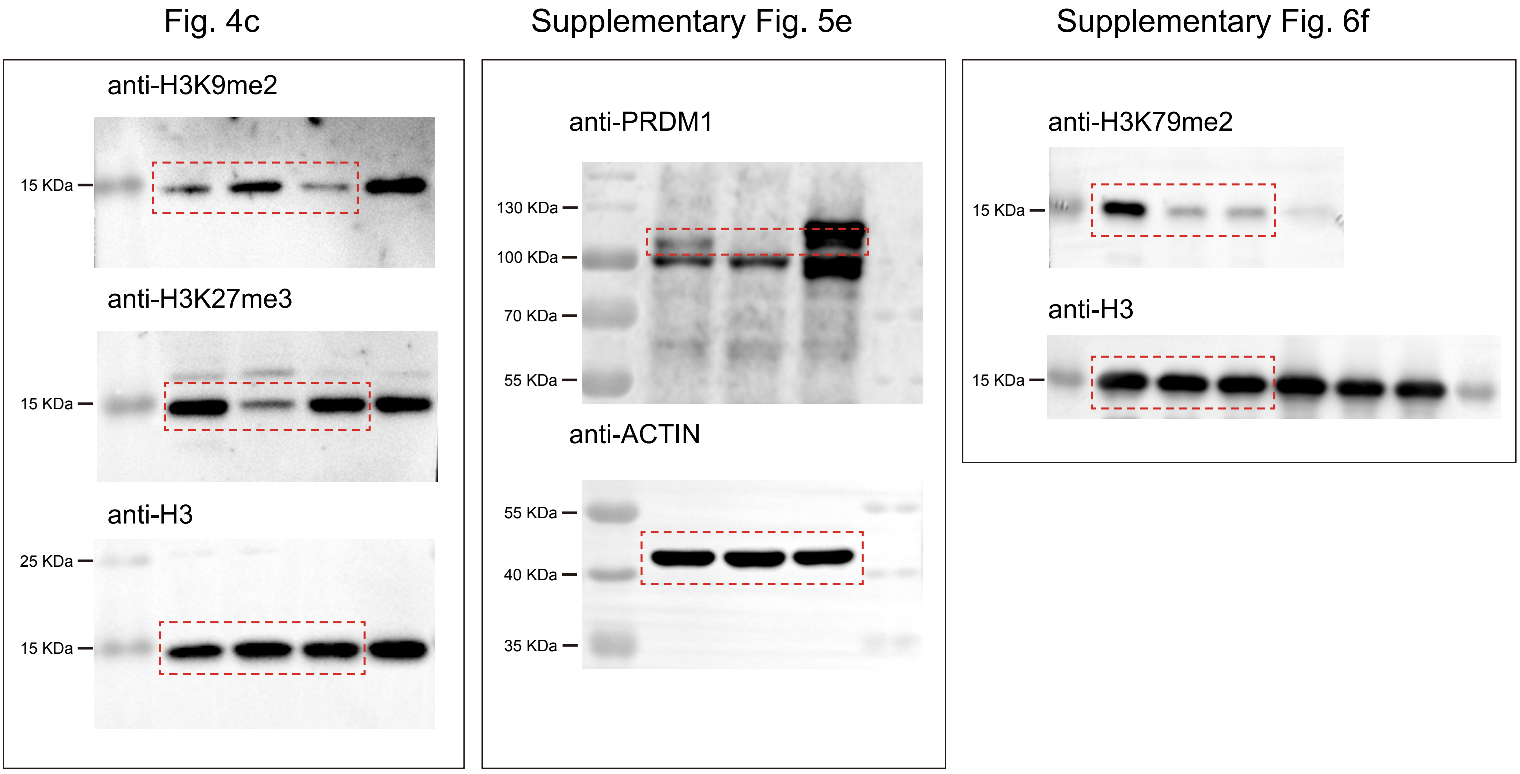

Supplementary Fig. 9 Uncropped western blot images
