# Supplementary material for: BMP4 drives primed to naïve transition through PGC-like state
Source: Nat Commun. 2022 May 19;13:2756. doi: 10.1038/s41467-022-30325-4 (PMC9120449; doi:10.1038/s41467-022-30325-4)
Supplement: Supplementary file 3 — Description of Additional Supplementary Files [file 41467_2022_30325_MOESM3_ESM.pdf]

Description of Additional Supplementary Files

File Name: Supplementary Data 1
Description: Primers for RT-qPCR used in this research.

File Name: Supplementary Data 2
Description: Primers for CRISPR/Cas9 used in this research.

File Name: Supplementary Data 3
Description: Primers for Bisulfite Sequencing used in this research.
