# Supplementary material for: BMP4 drives primed to naïve transition through PGC-like state
Source: Nat Commun. 2022 May 19;13:2756. doi: 10.1038/s41467-022-30325-4 (PMC9120449; doi:10.1038/s41467-022-30325-4)
Supplement: Supplementary file 7 — Reporting Summary [file 41467_2022_30325_MOESM7_ESM.pdf]

nature research

Corresponding author(s): Jing Liu, Duanqing Pei and Shangtao Cao

Last updated by author(s): Feb 17, 2021

Reporting Summary

Nature Research wishes to improve the reproducibility of the work that we publish. This form provides structure for consistency and transparency in reporting. For further information on Nature Research policies, see our Editorial Policies and the Editorial Policy Checklist.

Statistics

For all statistical analyses, confirm that the following items are present in the figure legend, table legend, main text, or Methods section.

n/a	Confirmed	
☐	☒	The exact sample size (*n*) for each experimental group/condition, given as a discrete number and unit of measurement
☐	☒	A statement on whether measurements were taken from distinct samples or whether the same sample was measured repeatedly
☐	☒	The statistical test(s) used AND whether they are one- or two-sided<br>*Only common tests should be described solely by name; describe more complex techniques in the Methods section.*
☒	☐	A description of all covariates tested
☐	☒	A description of any assumptions or corrections, such as tests of normality and adjustment for multiple comparisons
☐	☒	A full description of the statistical parameters including central tendency (e.g. means) or other basic estimates (e.g. regression coefficient) AND variation (e.g. standard deviation) or associated estimates of uncertainty (e.g. confidence intervals)
☐	☒	For null hypothesis testing, the test statistic (e.g. *F*, *t*, *r*) with confidence intervals, effect sizes, degrees of freedom and *P* value noted<br>*Give P values as exact values whenever suitable.*
☒	☐	For Bayesian analysis, information on the choice of priors and Markov chain Monte Carlo settings
☒	☐	For hierarchical and complex designs, identification of the appropriate level for tests and full reporting of outcomes
☒	☐	Estimates of effect sizes (e.g. Cohen's *d*, Pearson's *r*), indicating how they were calculated

Our web collection on statistics for biologists contains articles on many of the points above.

Software and code

Policy information about availability of computer code

Data collection	scRNA-seq and ChIP-seq libraries were subjected to pair-end sequencing on Illumina HiSeq X Ten and NovaSeq 6000, respectively; ATAC-seq and RNA-seq libraries were subjected to pair-end sequencing on Illumina Nextseq 500.
Data analysis	scRNA-seq, RNA-seq, ATAC-seq and ChIP-seq data were analyzed following the software documentations (Bowtie2,version 2.2.5; RSEM, version 2.4.1; DESeq2, version 1.22.1; EDASeq, version 2.4.1; goseq, version 1.22.0; MACS2, version 1.2.22; deeptools, version 2.5.4), no special changes. Microsoft Excel 2019 and GraphPad Prism 6 was used to analyze statistical data and draw graphs. FlowJo 10.4 was used to analyze the flow cytometry data. ImageJ 1.52 was used to analyze the bands intensity of western blot data.

For manuscripts utilizing custom algorithms or software that are central to the research but not yet described in published literature, software must be made available to editors and reviewers. We strongly encourage code deposition in a community repository (e.g. GitHub). See the Nature Research guidelines for submitting code & software for further information.

Data

Policy information about availability of data

All manuscripts must include a data availability statement. This statement should provide the following information, where applicable:

- Accession codes, unique identifiers, or web links for publicly available datasets
- A list of figures that have associated raw data
- A description of any restrictions on data availability

The scRNA-seq, RNA-seq, ATAC-seq and ChIP-seq data generated in this study have been deposited in the Gene Expression Omnibus (GEO) database under accession code GSE147088 [https://www.ncbi.nlm.nih.gov/geo/query/acc.cgi?acc=GSE147088]. Source data are provided with this paper. The source data underlying Figs. 2j, 3d, f, 4c, 5f, 6a, b, k, 7e and Supplementary Figs. 3b, 4a, 5d, i, 6a, c-e, k, 7d, f are provided as a Source Data file. The authors declare that all data

supporting the findings of this study are available within the article and its supplementary information files or from the corresponding author upon reasonable request.

Field-specific reporting

Please select the one below that is the best fit for your research. If you are not sure, read the appropriate sections before making your selection.

☒ Life sciences ☐ Behavioural & social sciences ☐ Ecological, evolutionary & environmental sciences

For a reference copy of the document with all sections, see nature.com/documents/nr-reporting-summary-flat.pdf

Life sciences study design

All studies must disclose on these points even when the disclosure is negative.

Sample size	No sample size calculation was performed. Sample size was chosen based on our experience and experiments from published literature (Yu et al, Nat Cell Bio, 2020).
Data exclusions	No data were exclude from the analysis.
Replication	For all the figures from which the significance were assessed, three biological replication experiments were preformed. The biological or technical replicates were present in relevant figure legends. The experimental findings were reliably reproduced.
Randomization	For transplantation experiments, Mice were selected at random for injection. For the statistics of immunostaining data, six random selected fields were analyzed. Randomization was not relevant to other experiments due to the use of cell lines.
Blinding	The investigates were blinded to group allocation during data collection and analysis.

Reporting for specific materials, systems and methods

We require information from authors about some types of materials, experimental systems and methods used in many studies. Here, indicate whether each material, system or method listed is relevant to your study. If you are not sure if a list item applies to your research, read the appropriate section before selecting a response.

Materials & experimental systems

n/a	Involved in the study
☐	☒ Antibodies
☐	☒ Eukaryotic cell lines
☒	☐ Palaeontology and archaeology
☐	☒ Animals and other organisms
☒	☐ Human research participants
☒	☐ Clinical data
☒	☐ Dual use research of concern

Methods

n/a	Involved in the study
☐	☒ ChIP-seq
☐	☒ Flow cytometry
☒	☐ MRI-based neuroimaging

Antibodies

Antibodies used	APC anti-c-Kit (eBioscience, 17-1171-83,1:200 for flow cytometry); Alexa Fluor 647 anti-mouse/human CD15 (SSEA-1) (Biolegend, 125607, 1:200 for flow cytometry); PE anti-mouse/rat CD61 (Biolegend, 104307, 1:500 for flow cytometry); anti-KLF4 (R&D system, AF3158, 1:100 for IF); anti-H3K9me2 (Abcam, ab1220, 1:100 for IF, 1:500 for western blot); anti-H3K27me3 (Merck, 17- 622, 1:200 for IF, 1:2000 for western blot); anti-PRDM1 (Invitrogen, 14-5963-82, 1:50 for IF, 1:100 for western blot); anti-ACTIN (Sigma, A5441, 1:4000 for western blot); anti-H3 (Abcam, ab1791, 1:2000 for western blot); anti-H3K79me2 (Active Motif, 39143, 1:1000 for western blot); anti-DDX4 (Abcam, ab13840, 1:400 for IF); anti-SOX9 (EMD Millipore, AB5535, 1:400 for IF); anti-γH2AX (Abcam, ab26350, 1:400 for IF); anti-SYCP3 (Abcam, ab15093, 1:400 for IF); anti-PNA (ThermoFisher, L21409 , 10 µg/ml for IF); anti-PLZF (Santa Cruz, sc-28319, 1:100 for IF)
Validation	APC anti-c-Kit: https://www.thermofisher.cn/cn/zh/antibody/product/CD117-c-Kit-Antibody-clone-2B8-Monoclonal/17-1171-83<br>Alexa Fluor 647 anti-mouse/human CD15 (SSEA-1): https://www.biolegend.com/en-us/products/alexa-fluor-647-anti-mouse-human-cd15-ssea-1-antibody-4819<br>PE anti-mouse/rat CD61: https://www.biolegend.com/en-us/products/pe-anti-mouse-rat-cd61-antibody-81<br>anti-KLF4: https://www.rndsystems.com/cn/products/mouse-klf4-antibody_af3158<br>anti-H3K9me2: https://www.abcam.cn/histone-h3-di-methyl-k9-antibody-mabcam-1220-chip-grade-ab1220.html<br>anti-H3K27me3: https://www.sigmaaldrich.cn/CN/zh/product/mm/17622<br>anti-PRDM1: https://www.thermofisher.cn/cn/zh/antibody/product/Blimp-1-Antibody-clone-6D3-Monoclonal/14-5963-82<br>anti-ACTIN: https://www.sigmaaldrich.cn/CN/zh/product/sigma/a5441<br>anti-H3: https://www.abcam.cn/histone-h3-antibody-nuclear-marker-and-chip-grade-ab1791.html<br>anti-H3K79me2: https://www.activemotif.com/catalog/details/39143/histone-h3-dimethyl-lys79-antibody-pab

anti-DDX4: https://www.abcam.cn/ddx4--mvh-antibody-ab13840.html
anti-SOX9: https://www.sigmaaldrich.cn/CN/zh/product/mm/ab5535
anti-γH2AX: https://www.abcam.cn/gamma-h2ax-phospho-s139-antibody-9f3-ab26350.html
anti-SYCP3: https://www.abcam.cn/scp3-antibody-ab15093.html
anti-PNA: https://www.thermofisher.cn/order/catalog/product/L21409?SID=srch-hj-L21409
anti-PLZF: https://www.scbt.com/p/plzf-antibody-d-9?requestFrom=search

Eukaryotic cell lines

Policy information about cell lines

Cell line source(s)	EpiSCs were derived from E5.5 mouse embryos generated by mating homozygous ΔPE-Oct4-GFP transgenic allele-carrying mice (CBA/CaJ X C57BL/6J) with 129/Sv female mice. BVSC-ESCs were obtained from the lab of Dr. Mitinori Saitou (Kyoto University). BVSC-EpiSCs were derived form the BVSC-ESCs.
Authentication	Cell lines were not authenticated.
Mycoplasma contamination	All of the cell lines have been confirmed as mycoplasma contamination free with the kit from lonza(LT07-318).
Commonly misidentified lines (See ICLAC register)	No commonly misidentified cell lines were used.

Animals and other organisms

Policy information about studies involving animals; ARRIVE guidelines recommended for reporting animal research

Laboratory animals	The mice used in this study were 8-week-old male ΔPE-Oct4-GFP transgenetic allele carrying mice (CBA/cAJ x C57bl/6J), female 129/Sv mice, female and male ICR mice, 10- to 15-day old W/Wv male mice.<br>The housing conditions for mice: 12 hrs light/12 hrs dark, 25 °C, 50% relative humidity.
Wild animals	This study did not involve wild animals.
Field-collected samples	This study did not involve samples collected from the field.
Ethics oversight	All the animal experiments were performed with the approval and according to the guidelines of the animal care and use committee of the Guangzhou Institutes of Biomedicine and Health.

Note that full information on the approval of the study protocol must also be provided in the manuscript.

ChIP-seq

Data deposition

☒ Confirm that both raw and final processed data have been deposited in a public database such as GEO.

☒ Confirm that you have deposited or provided access to graph files (e.g. BED files) for the called peaks.

Data access links<br>*May remain private before publication.*	All sequencing data that support the findings of this study have been deposited in NCBI's Gene expression Omnibus (GEO) under accession code GSE147088.
Files in database submission	H3K79me2-EpiSC, H3K79me2-D3(-DOT1Li), H3K79me2-D3(+DOT1Li), H3K27me3-EpiSC, H3K27me3-D3(-DOT1Li), H3K27me3-D3(+DOT1Li).
Genome browser session (e.g. UCSC)	IGV

Methodology

Replicates	There were no biological replicates. The ChIP-seq experiment was performed once.
Sequencing depth	Raw reads of all samples above have been deposited in NCBI's Gene expression Omnibus (GEO) under accession number GSE147088 where the sequencing depth information is available.
Antibodies	anti-H3K79me2 (Active Motif, 39143); anti-H3K27me3 (CST, 9733S); spike-in antibody (Active motif, 61686)
Peak calling parameters	Peaks were generated by MACS2 (v 2.1.2) with the default parameters.
Data quality	The default cutoff of MACS2 were used for the significantly enriched peaks.
Software	Bowtie2 (v 4.1.2), MACS2 (v 2.1.2), deeptools (v 2.5.4)

Flow Cytometry

Plots

Confirm that:

☒ The axis labels state the marker and fluorochrome used (e.g. CD4-FITC).

☒ The axis scales are clearly visible. Include numbers along axes only for bottom left plot of group (a 'group' is an analysis of identical markers).

☒ All plots are contour plots with outliers or pseudocolor plots.

☒ A numerical value for number of cells or percentage (with statistics) is provided.

Methodology

Sample preparation	Cells were dissociated with 0.05% trypsin-EDTA and neutralized with DMEM containing 10% FBS. After the dissociation, cells were collected by centrifugation and resuspended with flow cytometry buffer (DPBS with 0.1% BSA and 1 mM EDTA) at a density of one million cells per 100 µl buffer. Cells were then incubated with fluorophore- conjugated antibodies for 30 min at 4 °C. After incubation, cells were washed with DPBS for three times, resuspended with flow cytometry buffer and filtered with 40 µm cell strainer to remove large clumps of cells.
Instrument	BD Accuri C6 Plus; BD LSRFortessa X-20; BD FACS Arial III.
Software	FlowJo software
Cell population abundance	A minimum of 10,000 cells were counted per sample analyzed.
Gating strategy	We set the preliminary FSC/SSC gate to remove debris, and single cell gate to select for single cells, then we set the gate based on the fluorescence negative control. Please see an example in Supplementary Fig. 8.

☒ Tick this box to confirm that a figure exemplifying the gating strategy is provided in the Supplementary Information.
